# The Rapid Diagnosis of Rabies

**Published:** 1900-07

**Authors:** Mazyck P. Ravenel, D. J. McCarthy

**Affiliations:** The Laboratory of the State Live Stock Sanitary Board of Pennsylvania and the Pepper Clinical Laboratory, University of Pennsylvania; The Laboratory of the State Live Stock Sanitary Board of Pennsylvania and the Pepper Clinical Laboratory, University of Pennsylvania


### THE RAPID DIAGNOSIS OF RABIES. PRELIMINARY REPORT.[1]

By Mazyck P. Ravenel, M.D., and

D. J. McCarthy, M.D.

(*From the Laboratory of the State Live Stock Sanitary Board of Pennsylvania and the Pepper Clinical Laboratory, University of Pennsylvania.*)

Before taking up the newer methods of diagnosis of rabies, as suggested by Babès, and later by Van Gehuchten and Nélis, it will be well to review the work which lead to the development of these methods. The work of Baltzer calling attention to the changes in the central nervous system was followed

[1] Read before the Pathological Society of Philadelphia, June 14, 1900.

in 1874 by Benedikt's article, in which he described perivascular infiltration of leucocytes and erythrocytes. Kolesnikoff, in 1876, called attention to an invasion of the pericellular spaces by "indifferent round-cells" affecting the hemispheres, cerebellum, spinal cord, and the intervertebral ganglia. Schaffer likewise made a very careful study of the vascular and cellular changes, and called attention to "a hyaline and fibrillar degeneration and vacuolation of the anterior horn cells of the cord."

Nothing of practical importance resulted from these studies until Babès, in 1892, published the results of his investigations in this field. He confirmed the work of Balzer and Kolesnikoff, described in detail the changes in the ganglion cells of the gray matter, and especially of the bulbar nuclei, and added what he considered to be the typical and characteristic lesions. They consisted of pericellular accumulations of embryonal cells, to which he gave the name "tubercles rabiques." The cells of the bulbar nuclei degenerate and present the various stages of chromatolysis. While this process is going on numerous nuclei are seen about the cell, and when the cell is completely degenerated these occupy the cell-area and constitute the "tubercle rabique." Babès, therefore, believes that microscopical examination of the cord furnishes us with the best means for the rapid diagnosis of rabies. Except in Babès own laboratory but little use has been made of this method.

Recently Nélis, working with Van Gehuchten, discovered in the spinal ganglia of two men dead of rabies, and in many of the lower animals, peculiar changes. Normally the intervertebral ganglion is composed of a network of supporting tissue holding in its meshes the nerve cells, enclosed in endothelial capsules. In the rabid animal an infiltration of leucocytes takes place in the stroma, and a proliferation of the endothelial cells of the capsule takes the place of the degenerating nerve cell. These changes are widespread and constant. There are but few unaffected capsules, and in advanced cases the section has very much the appearance of an alveolar sarcoma. Van Gehuchten and Nélis question the value of Babès' findings. They consider his cellular changes as secondary, and the rabic tubercle as inconstant and of little value for diagnostic purposes.

With a view of determining what of value there might be in these different methods, and to what practical use they could be put in our daily laboratory work, we have examined the cord, medulla, and intervertebral ganglia of a number of cases which have come to us for diagnosis. In this preliminary report six cases are detailed. By the fall meeting of the society we expect to be able to report more fully, and to give the results of a number of control experiments on this interesting subject.

Of the six cases studied, two were dogs and four rabbits. The medulla was hardened in formalin (10 per cent.), then changed successively to 95 per cent. and absolute alcohol. The tissues were cut for the most part without embedding, though celloidin was used in some cases. In all cases the changes noted in the intervertebral ganglia were constant, and corresponded to those described by Van Gehuchten and Nélis. Chromatolytic changes were present in all the cells when stained by the Nissl method, but the capsular changes were best seen in sections stained by hæmatoxylin and eosin. Since these latter changes are the essential diagnostic features of the sections, we would suggest that material unfit for the Nissl method would still show the capsular changes when stained by hæmatoxylin and eosin.

The ganglia from the rabbits showed the most advanced changes. The disease in them was experimentally produced by subdural inoculation with material from dogs. In both dogs distinct diagnostic changes had taken place in the cells and their capsules. The changes in the cells are as follows: A diffuse chromatolysis affecting all the cells first occurs, followed by a retraction of the cell from the capsule, and a proliferation of the cells of the capsule itself. These newly formed cells press on the degenerating ganglion cell, and its complete destruction is followed by the filling up of the capsule by the new cells. This stage was only occasionally seen in the ganglia from the dogs, there being usually a diffuse chromatolysis with proliferation of the capsular cells, not, however, to a sufficient extent to fill up the capsule and obliterate all trace of the ganglion cells.

We have examined also the medulla in all the cases for the rabic tubercles of Babès. They were present in five of the six; but the method is not so easily carried out, nor are the lesions so apparent on a rapid examination as in the method described by Nélis.

